# The effect of recombinant human epidermal growth factor on radiation dermatitis in rectal and anal cancer patients: a self-controlled study

**DOI:** 10.1186/s12885-022-10226-x

**Published:** 2022-11-05

**Authors:** Shuai Liu, Yun-Long Wang, Shu-Ting Shi, Guang-Dong Zeng, Yi-Wen Song, Xiao-Dong Zhang, Jian Zheng, Xin-Juan Fan, Yan-Ping Liu

**Affiliations:** 1 Department of Radiation Oncology, The Sixth Affiliated Hospital, Sun Yat-sen University, Guangzhou, 510655 People’s Republic of China; 2 Guangdong Provincial Key Laboratory of Colorectal and Pelvic Floor Diseases, The Sixth Affiliated Hospital, Sun Yat-sen University, Guangzhou, 510655 People’s Republic of China; 3 Clinical Medicine College, Tianjin Medical University, Tianjin, 300276 People’s Republic of China; 4 Department of Pathology, The Sixth Affiliated Hospital, Sun Yat-sen University, Guangzhou, 510655 People’s Republic of China

**Keywords:** rhEGF, Radiation dermatitis, Rectal and anal cancer, Clinical trial

## Abstract

**Background:**

Our previous study reported that recombinant human epidermal growth factor (rhEGF)-triggered EGFR internalization promoted radioresistance. Here, we aimed to evaluate the effect of rhEGF on the skin protection of rectal and anal cancer patients receiving radiotherapy.

**Methods:**

One hundred and ninety-three rectal and anal cancer patients who received radiotherapy were prospectively enrolled from January 2019 to December 2020. To perform self-controlled study, the left and right pelvic skin area (separated by midline) were randomly assigned to the rhEGF and control side. The association between radiation dermatitis and factors including rhEGF, the dose of radiotherapy and tumor distance from anal edge were analyzed.

**Results:**

Among 193 enrolled patients, 41 patients (21.2%) did not develop radiation dermatitis, and 152 patients (78.8%) suffered radiation dermatitis on at least one side of pelvic skin at the end of radiotherapy. For the effect on radiation dermatitis grade, rhEGF had improved effect on 6 (4.0%) patients, detrimental effect on 2 (1.3%) patients, and no effect on 144 (94.7%) patients. Whereas for the effect on radiation dermatitis area, rhEGF showed improved effect on the radiation dermatitis area of 46 (30.2%) patients, detrimental effect on 15 (9.9%) patients, and no effect on 91 (59.9%) patients. The radiation dermatitis area of rhEGF side was significantly smaller than that of control side (*P* = 0.0007).

**Conclusions:**

rhEGF is a skin protective reagent for rectal and anal cancer patients receiving radiotherapy.

**Trial registration:**

Chinese Clinical Trial Registry identifier: ChiCTR1900020842; Date of registration: 20/01/2019.

## Introduction

Worldwide, about 300,000 cancer patients receive pelvic radiotherapy every year [1], including rectal and anal cancer patients. Patients with locally advanced rectal cancer need to receive preoperative neoadjuvant radiotherapy to obtain better local control rate [2] and achieve long-term survival through radical radiotherapy [3]. Radiotherapy plays an indispensable role in these patients, but it also brings potential complications, such as radiation-induced skin reaction or radiation dermatitis. In the process of radiotherapy, radiation injury usually occurs in perianal, perineal and inguinal skin due to the formation of physical effect caused by skin wrinkles at these sites. Radiation dermatitis is a very common radiation injury from radiotherapy for rectal and anal cancer patients. More than half of rectal and anal cancer patients will develop varying degrees of radiation dermatitis during radiotherapy [4, 5]. Patients with anal cancer may have more severe skin toxicity due to a higher dose delivering to the skin [6]. radiation induced severe skin reaction can lead to local or systemic infection. Patients with radiation dermatitis exceeding Radiation Therapy Oncology Group (RTOG) grade III or above would cause severe pain. The patients' physical pain and psychological impact could affect the therapy result and the quality of life.

Our previous study reported recombinant human epidermal growth factor (rhEGF)/EGFR enhanced radioresistance [7]. Actually, in some previous studies, rhEGF was reported to be beneficial for radiation induced skin injury [8, 9] in other kinds of tumors. However, the quality of these evidence was not high, and whether rhEGF was useful in patients with rectal and anal cancer remained to be evaluated. Here, we conducted a self-controlled study to provide high-level evidences for the application of rhEGF in the skin protection of rectal and anal cancer patients receiving radiotherapy.

## Methods

### Patients

**Fig. 1:**
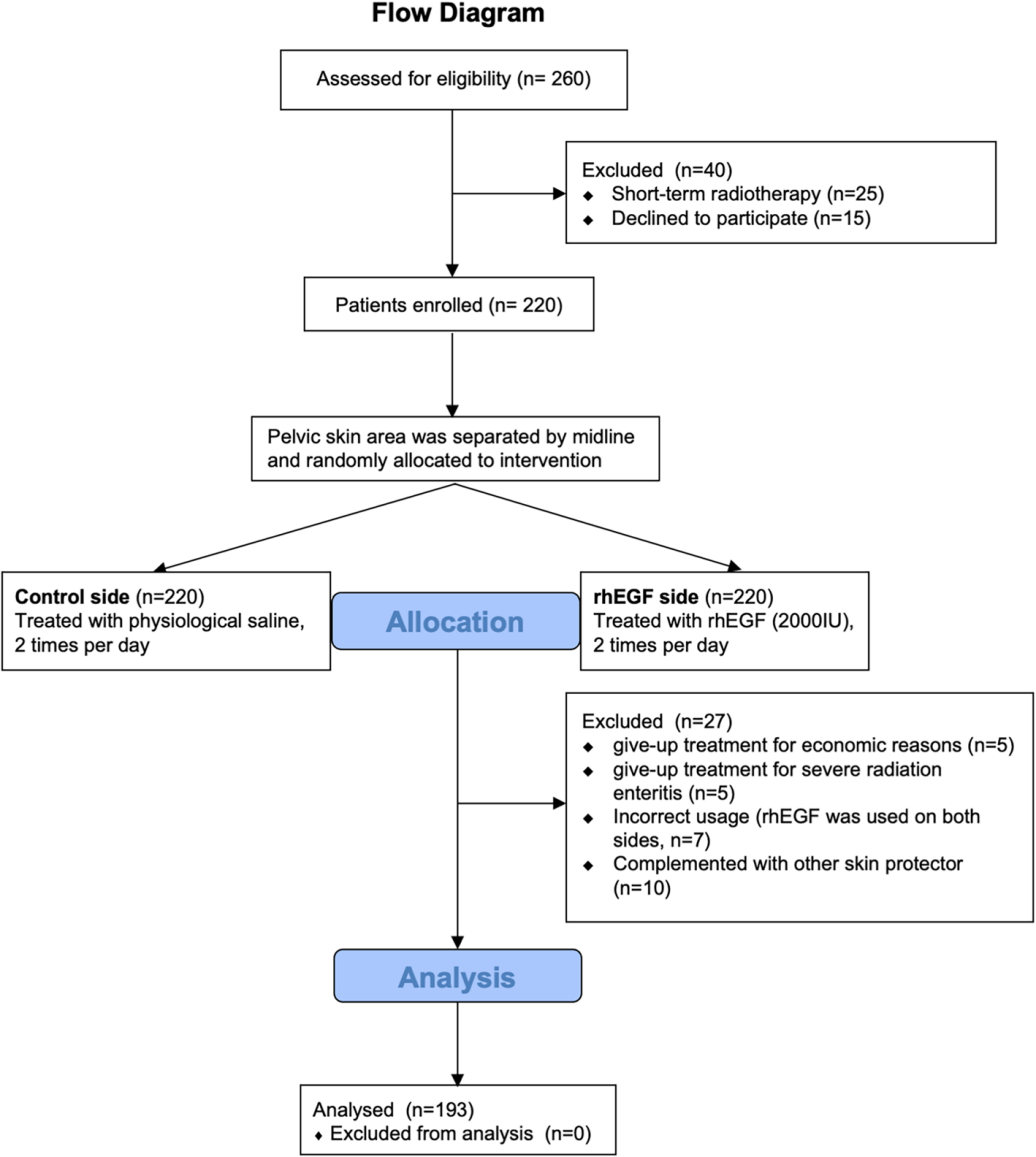
Consolidated Standards of Reporting Trials (CONSORT) flowchart

This study is a self-controlled study. This study was approved by the Ethics Committee of the Sixth Affiliated Hospital of Sun Yat-sen University (Ethics number: 2018ZSLYEC-116). Rectal and anal cancer patients who received and completed pelvic intensity modulated radiotherapy were enrolled from January 2019 to December 2020. The enrolled flow diagram is shown in Fig. 1. All patients involved had complete medical data during treatment. All methods were carried out in accordance with relevant guidelines and regulations. Informed consent was obtained from all subjects.

### Radiation therapy

All patients underwent radiation therapy: Briefly, all enrolled patients received VMAT with an Elekta Synergy accelerator (with 80 MLCs) with 6MV photon, delivered at 1.8–2.0 Gy per day per fraction from Monday to Friday for a total of 25–32 fractions and a total dose of 45.0–64 Gy. Monaco (version 9.0) was used for treatment planning. In Monaco (Version: 5.11.03), a collapsed cone convolution superposition model is used. The total dose of radiotherapy of all patients were converted into equivalent biological effective dose (BED) according to the formula BED = Nd (1 + d/ (α / β)) (N is the number of fractions, d is the dose per fraction and α / β = 10 was assumed to be 10 for tumor) to ensure the comparability of data [10]. The median BED was 60 (53–76.8) Gy. During radiotherapy, all patients received combined chemotherapy synchronously and different chemotherapy regimens were not the criteria for patient recruitment.

### Randomization and treatment

**Fig. 2:**
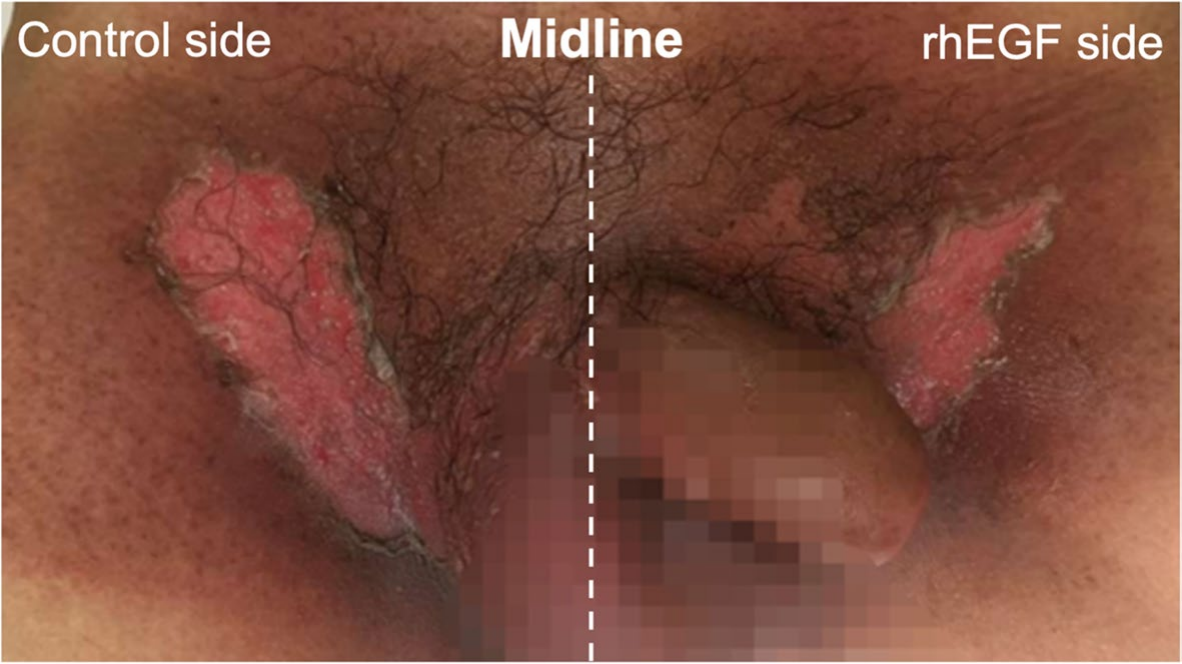
An example radiation dermatitis. The left and right sides were all classified as radiation dermatitis grade 2 for the appearance of bright red erythema, flaky wet desquamation according to RTOG system. The area of radiation dermatitis was scored 1 point on the left and 2 points on the right of the body according to the palm five-point scale

The pelvic skin area receiving radiotherapy was divided into two sides separated by the midline (Fig. 2), and the rhEGF side and control side were randomly assigned by the blinded draw, each side is an independent object of study. Self-controls were formed on both sides. The skin condition in perianal, perineal and inguinal areas was observed. Firstly, the rhEGF side and the control side were all cleaned with physiological saline twice a day by the same nurse every day from the beginning of radiotherapy. After cleaning, the rhEGF side was sprayed with rhEGF twice a day, 2000 IU for each time, once at one hour after radiotherapy and once again before going to bed at night. The skin of control side was sprayed with the same volume of physiological saline. For EGF or physiological saline treatment, the distance from the handheld bottle to the skin was 10 cm, and 5 cm^2^ of skin was sprayed with a dose of 200 IU/spray, for a total of 10 times.

### Scoring of radiation dermatitis grade and area

From the beginning to the end of radiotherapy, the grade of radiation dermatitis was blindly evaluated every week by two radiation oncologists who did not know the rhEGF side and the control side. The evaluation of radiation dermatitis is based on the radiodermatitis acute radiation injury classification of RTOG 4.0 [11]: Grade 0: no change; grade 1: follicular dark red / alopecia / dry peeling / reduced sweating; grade 2: tenderness or bright red erythema, flaky wet desquamation / moderate edema; grade 3: fusion wet desquamation outside skin folds, or sunken edema; grade 4: ulcer, bleeding, or necrosis.

At the end of radiotherapy, the area (A) of pelvic radiation dermatitis was evaluated according to the palm five-point scale: A ≤ 1/5 palm, 1 point; 1/5 < A ≤ 2/5 palm, 2 points; 2/5 < A ≤ 3/5 palm, 3 points; 3/5 < A ≤ 4/5 palm, 4 points; 4/5 < A ≤ 1 palm, 5 points; less than 1 point range, 0.5 points. The higher score represents the larger area of radiation dermatitis. If the radiation dermatitis grade or area assessments from two doctors were inconsistent, the doctors were asked to negotiate to give a final assessment. For the results of rhEGF treatment referring to radiation dermatitis grade or area score, improved effect means rhEGF side < control side, whereas detrimental effect means rhEGF side > control side.

### Statistical analysis

SPSS version 23.0 for Windows (IBM Corporation, Armonk, NY, USA) was used for statistical analysis. The difference on weekly radiation dermatitis grade between rhEGF side and control side was compared with the Chi-square test (two-side). The difference on radiation dermatitis area between rhEGF side and control side was compared with paired t-test (two-side). The association between radiation dermatitis grade/area and different clinical characteristic was analyzed using Chi-square test. *P* < 0.05 was considered as significantly different.

## Results

### Patients’ clinical characteristics

A total of 260 patients with rectal and anal cancer were prospectively assessed for eligibility from January 2019 to December 2020. Twenty-five patients receiving short-term radiotherapy were excluded because they didn’t meet the eligibility. Fifteen patients declined to participate. From 260 patients assessed, 220 patients were enrolled in this study. During radiotherapy, 10 patients gave up the treatment for economic reasons (5 patients) or suffering severe radiation enteritis (5 patients). Seventeen patients were excluded because the incorrect usage of rhEGF solution (7 patients) or receiving other skin-protection treatment (10 patients). Finally, 193 patients completed the treatment and were enrolled for data analysis (Fig. 1).

The mean age of 193 patients was 54.1 (16–80) years (Table 1). The mean distance from tumor to anal verge was 4.4 (0.1–15.0) cm. Thirty-three patients got a colostomy. The mean biological effective dose and fractionated dose was 58.9 (39.0–76.8) Gy and 1.96 (1.36–3.00) Gy, respectively. The median fraction number of radiotherapy for the first appearance of radiation dermatitis was (15–17) times, and the median radiation dose was (30–34) Gy.

**Table 1:** The association between clinical characteristics and radiation dermatitis on the control side

		Total	RD grade			RD area score		
			0-I	II-IV	*P* ^a^	≤ 2	> 2	*P* ^a^
Gender					0.050			0.081
	Male	111	83	28		71	40	
	Female	82	51	31		48	34	
Age (years)					0.019			0.037
	≤ 55	101	76	25		67	34	
	> 55	92	58	34		52	40	
Tumor distance from anal verge					0.002			< 0.001
	≤ 4	104	66	38		52	52	
	> 4	89	68	21		67	22	
Colostomy ^b^					0.291			0.002
	+	33	24	9		28	5	
	-	160	110	50		91	69	
Biological effective dose (Gy)					< 0.001			< 0.001
	≤ 60	167	124	43		110	57	
	> 60	26	10	16		9	17	
Fractionated dose (Gy)					0.003			0.012
	≤ 2	174	126	48		111	63	
	> 2	19	8	11		8	11	
Chemotherapy					0.216			1.000
	5FU/Capecitabine	7	6	1		5	2	
	FOLFOX	171	120	51		101	70	
	XELOX	4	4	0		3	1	
	MMC + 5FU	11	4	7		4	7	

^a^ *P* values were calculated with chi-square test

^b^ -, absence; + , presence

### Skin reaction

At the end of radiotherapy, 41 patients (21.2%) did not develop radiation dermatitis, and 152 patients (78.8%) suffered radiation dermatitis on at least one side of the rhEGF side or the control side (Table 2). In the patients with radiation dermatitis, the median fraction number of radiotherapy for the first appearance of radiation dermatitis was 17 (5–31), and the median radiation dose for the first appearance was 34 Gy (10–64) (inguinal skin) and 32 Gy (10–64) (perineal skin). In all patients, no allergic reaction related to rhEGF was found. The significant associations between radiation dermatitis and patients’ characteristics, including age, tumor distance from anal verge, colostomy, BED and fractionated dose were found (Table 1).

**Table 2:** The radiation dermatitis grade at every week post radiotherapy

RD grade	1^st^ week			2^nd^ week			3^rd^ week			4^th^ week			End of radiotherapy ^a^		
	rhEGF	Control	*P*	rhEGF	Control	*P*	rhEGF	Control	*P*	rhEGF	Control	*P*	rhEGF	Control	*P* ^b^
0	187	186	0.778	150	137	0.130	110	102	0.453	69	68	0.309	43	41 ^c^	0.889
I	6	7		43	56		83	90		105	95		95	93	
II	0	0		0	0		0	1		19	29		39	45	
III	0	0		0	0		0	0		0	1		16	14	
IV	0	0		0	0		0	0		0	0		0	0	
Total	193	193		193	193		193	193		193	193		193	193	

^a^ Radiotherapy was completed at/after the 5^th^ week

^b^ *P* values were calculated using chi-square test

^c^ Radiation dermatitis wasn’t presented on either control or rhEGF side of these 41 patients

### The effect of rhEGF

Two outcomes were evaluated in this study: radiation dermatitis grade and radiation dermatitis skin area.

The weekly evaluation of radiation dermatitis grade was shown in Table 2. There was no significantly difference regarding to radiation dermatitis grade between rhEGF side and control side in the whole radiotherapy process. In 152 patients suffering radiation dermatitis at the end of radiotherapy, rhEGF had improved effect on 6 (4.0%) patients, detrimental effect on 2 (1.3%) patients, and no effect on 144 (94.7%) patients (Table 3). These results suggest that rhEGF had no impact on the radiation dermatitis grade.

**Table 3:** The effect of hrEGF on radiation dermatitis

	RD (hrEGF *vs* Ctrl)			Total
	Improved effect	Detrimental effect	No change	
RD grade	6 (4.0%)	2 (1.3%)	144 (94.7%)	152
RD area score	46 (30.2%)	15 (9.9%)	91 (59.9%)	152

**Fig. 3:**
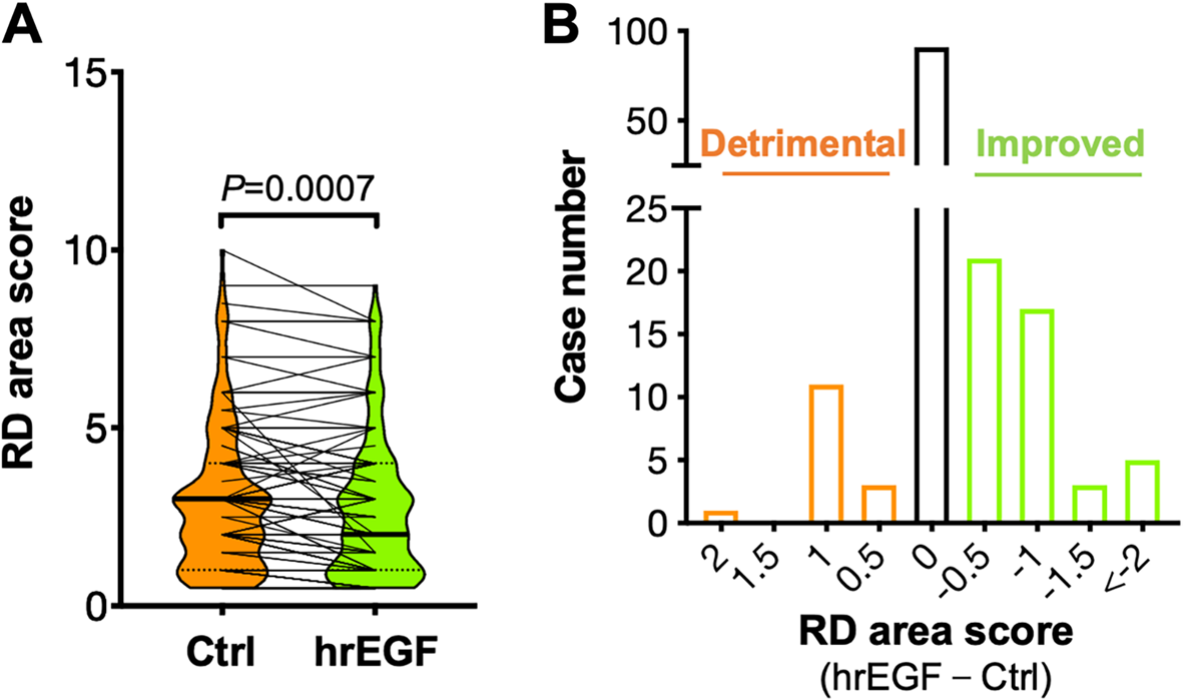
rhEGF treatment reduced the radiation dermatitis area of patients. **A**, the difference of RD area between rhEGF and control side on 152 patients were compared using paired t-test. **B**, the area changes resulting from rhEGF treatment. The X axis showed the difference in RD area score (rhEGF – Control)

The radiation dermatitis area was evaluated at the end of radiotherapy. In 152 patients suffering radiation dermatitis, rhEGF showed improved effect on the radiation dermatitis area of 46 (30.2%) patients, detrimental effect on 15 (9.9%) patients, and no effect on 91 (59.9%) patients (Table 3). The median score of radiation dermatitis area on rhEGF side was 2 (0.5–9.0), and that of the control side was 3 (0.5–10.0). Statistical analysis (paired *t*-test) showed that the radiation dermatitis area of rhEGF side was significantly smaller than that of control side (*P* = 0.0007) (Figs. 2 and 3A-B). These data indicated that rhEGF treatment could reduce the radiation dermatitis area of rectal and anal cancer patients.

## Discussion

The severity of radiation dermatitis was correlated with external factors (dose, exposure area, etc.) and internal factors (age, sex, nutritional status, etc.) [12]. Bias will arise due to different internal and external factors if we make a comparative between different patients. Therefore, this study is designed as a self-controlled study to eliminate external factors as much as possible, comparing the left and right sides of each patient. For the skin area received radiotherapy of rectal and anal cancer are symmetrical due to the symmetric target area in these patients, self-comparison taking the midline as the dividing line can well balance the uneven brought by a series of factors, such as age, sex and so on. However, the dose distribution between the left and right sides could be asymmetrical for anal cancer patients with inguinal lymph nodes, as the side containing lymph nodes often received a higher dose. After the balance of influencing factors, the effect of treatment medicine of dermatitis will be better reflected.

Epidermal growth factor (EGF) is a polypeptide containing 53 amino acids [13]. EGF helps maintain homeostasis by regulating the proliferation, growth and migration of epithelial cells. rhEGF can also induce angiogenesis and provide nutritional support for tissue. Our previous study showed that EGF-triggered epidermal growth factor receptor (EGFR) internalization promoted radiation-induced DNA double-strand breaks [7]. Therefore, EGF may protect skin from radiation-induced skin injury by enhancing DNA damage repair and reducing radiation-induced cell death. Additionally, EGF has been shown to protect skin from radiation injury by promoting the proliferation of fibroblasts, epidermal stem cells, and keratinocytes [14, 15].

The purpose of this study was to observe the effectiveness of rhEGF for skin protection in patients with rectal and anal cancer during radiotherapy and to investigate factors associated with radiation dermatitis. The results showed that the incidence of radiation dermatitis for rectal and anal carcinoma patients receiving radiotherapy was 78.8%. Elder age, lower tumor location, no colostomy, higher biological effective dose and higher fractionated dose were all related to the development of radiation dermatitis during radiotherapy. RhEGF is a kind of medicine that is economical, easy to use with high compliance and could effectively reduce the area of radiation dermatitis. This study provides high-level evidence-based medical evidence for the application of rhEGF in skin protection of rectal and anal cancer during radiotherapy.

Pelvic tumors are often accompanied by radiation dermatitis when the tumor of the pelvis is irradiated by external radiation. The maximum dose delivered by modern radiotherapy equipment is often deposited subcutaneously at 0.5-4 cm. Dry peeling can occur after irradiation with 10 Gy. 15 Gy often causes wet peeling, accompanied by varying degrees of radiation mucosal damage, skin damage, pigmentation and other reactions. In 20–30 Gy, patients often have erythema, itching, dry peeling, severe skin blisters, epidermis exfoliation, exudate and ulcers, causing local or systemic infection, or even interrupting radiotherapy, affecting the effect of treatment [16]. Radiation injury of perianal skin is a common complication after pelvic radiotherapy. Due to the particularity of anorectal function and location, the perianal skin of the patients is prone to suffer infection, which further leads to the difficulty of wound healing and makes the patients suffer a great deal of pain.

The drugs to relieve radiation dermatitis include Essex cream, Calendula Weleda, glycerine cream and so on. RhEGF is one of the drugs for the prevention and treatment of radiation dermatitis [17, 18]. HONG and Lee suggested that rhEGF has a good effect on the prevention and treatment of oral mucositis in patients with head and neck tumors [19, 20]. KONG et al. found that it can be used in radiotherapy for patients with breast cancer. RhEGF can reduce the occurrence of radiation dermatitis and relieve the pain of patients [21]. In the current study, we also found that rhEGF can reduce the area of radiation dermatitis in patients with rectal and anal carcinoma during radiotherapy. At the end of radiotherapy, the RTOG grading of radiation dermatitis was not significantly reduced, while the area of radiation dermatitis was reduced. This may be related to the relative high radiation dose. In fact, it shows that although rhEGF cannot counteract and reverse the skin reaction caused by radiation dose, it could relieve the pain of radiotherapy and accelerate the healing of skin to some extent. Besides, different types of dressing have been shown as effective ways to prevent and treatment of radiation dermatitis [22]. A combination between rhEGF and dressing may be an efficient treatment, which could be studied in further assessment.

It was easily explained that the distance between the lower edge of the tumor and the perianal skin was related to the severity of radiation dermatitis. The closer target irradiation area from the anus, skin around the anus more prone to produce radioactive damage. The area of radiation dermatitis in patients with low tumor is about 3.5 times larger than that in patients with high tumor. Some patients had temporary colostomy due to obstruction before radiotherapy. After temporary colostomy, the patient's excretion is excreted only a small amount through the anus or not through the anus at all, which largely ensures the cleanliness of the perianal skin and reduces the area of radiation dermatitis. Therefore, patients with colostomy tend to have a mild degree of radiation dermatitis. The level of dose will affect the severity of radiation dermatitis, which is a more accepted view in many studies [23–25]. Behroozian et al. found that in breast cancer, radiation dose is a predictor of the severity of radiation dermatitis. Dose more than 50 Gy/25f is related to pain, and dose boost is the only factor related to bleeding [23]. Therefore, we should pay more attention to proper patient education and early prevention in patients with low tumor, receiving high dose of radiotherapy and without colostomy, who will be high-risk groups of severe dermatitis of rectal and anal cancer.

There are shortcomings in the present study. Firstly, as patients were usually discharged at the end of radiotherapy, so we did not observe and record the skin condition of patients after discharge. This may result in the lack of some important information because in some patients the most prominent radiation dermatitis occurs shortly after the end of irradiation. Consistently, the effect of rhEGF on the healing of radiation dermatitis remains to be further investigated, which needs a longtime of rhEGF treatment and follow-up after radiotherapy. Besides, we noticed the weakness of our study that the area of radiation dermatitis was only assessed at the end of radiotherapy, but not evaluated weekly, therefore leading to the absence of the data of continuous observation. Additionally, as the accurate data of the area/volume of irradiated skin which received at least 10, 30 or > 50 Gy in each patient is unavailable, we did not analyze the effect of radiation dose or the area/volume of the irradiated skin on radiation dermatitis, which could be the most objective factor related to radiation dermatitis.

In conclusion, our data showed that rhEGF is a skin protective reagent for rectal and anal cancer patients receiving radiotherapy.

## Conclusion

rhEGF is a skin protective reagent for rectal and anal cancer patients receiving radiotherapy.

## Data Availability

The datasets generated and/or analysed during the current study are not publicly available due that these data will be prepared for further studies. The relevant data will be released when all the studies are completed, but are available from the corresponding author on reasonable request.
